# Cancer risk in East Asian patients associated with acquired haemolytic anaemia: a nationwide population-based cohort study

**DOI:** 10.1186/s12885-016-2098-3

**Published:** 2016-02-04

**Authors:** Victor C. Kok, Fung-Chang Sung, Chia-Hung Kao, Che-Chen Lin, Chun-Hung Tseng

**Affiliations:** Division of Medical Oncology, Department of Internal Medicine, Kuang Tien General Hospital, Taichung, 43303 Taiwan; Department of Biomedical Informatics, Asia University, Taichung, 41354 Taiwan; Department of Health Services Administration, China Medical University, Taichung, 40402 Taiwan; Department of Nuclear Medicine and PET Centre, China Medical University Hospital, Taichung, 40402 Taiwan; Management Office for Health Data, China Medical University Hospital, Taichung, 40402 Taiwan; Department of Neurology, China Medical University Hospital, Taichung, 40447 Taiwan

**Keywords:** Anaemia, Haemolytic, Causality, Non-autoimmune haemolytic anaemia, Retrospective cohort study, Population-based study

## Abstract

**Background:**

This study investigated whether patients with acquired haemolytic anaemia (AHA) would have elevated cancer risk including that for non-haematological solid tumours. We further examined whether the cancer risk would be different between patients with autoimmune type AHA (AIHA) and patients of non-AIHA.

**Methods:**

Using nationwide population-based insurance claims data of Taiwan we identified a cohort of patients with AHA with no pre-existing cancer, (*n =* 3902) and a comparison cohort (*n =* 39020) without AHA, frequency-matched by gender, age, urbanization of residency and diagnosis date. Incidence and Cox method estimated adjusted hazard ratios (aHR) of cancers controlling covariates by the end of 2010 were calculated. Risks between patients with AIHA and non-AIHA were compared. Sensitivity analysis was carried out to measure the risk of cancer between patients with and without AHA by follow-up years.

**Results:**

Patients with AHA had a 90 % greater incidence of cancer than controls, with an aHR of 1.78 (95 % confidence interval (CI), 1.50–2.12)]. The overall aHRs of cancer for patients with AIHA and non-AIHA were 2.01 (95 % CI, 1.56–2.59) and 1.87 (95 % CI, 1.53–2.29), respectively, compared with the comparison cohort. The aHRs for lymphatic-haematopoietic malignancy were 19.5 and 9.59 in the AIHA and non-AIHA cohorts, respectively. No hazard of colorectal, lung, liver or breast cancer was significant.

**Conclusions:**

There is a near 2-fold elevated risk for subsequent cancer in patients with AHA, particularly for lymphatic-haematopoietic malignancy, which is much greater for patients with AIHA than non-AIHA. These findings can help clinicians decide patient-centred personalized long-term management.

## Background

Acquired haemolytic anaemia (AHA) is the second most prevalent haemolytic anaemia in clinical medicine after sickle cell anaemia. With respect to mechanisms, AHA can be classified on the basis of its pathogenesis: haemolysis due to intracorpuscular defects or extracorpuscular factors. Paroxysmal nocturnal haemoglobinuria (PNH) is the only known AHA due to intracorpuscular defects caused by an acquired somatic mutation. Exogenous extracorpuscular factors that can cause haemolytic anaemia include autoimmune and non-autoimmune factors such as mechanical destruction, exposure to a toxic agent or drug and infections. Autoimmune haemolytic anaemia (AIHA) is the most common form of AHA in the world, excluding regions where malaria is endemic.

Recent studies have linked acquired idiopathic autoimmune haemolytic anaemia to an increased risk for future haematolymphoproliferative malignancy [1–6]. A pooled analysis of self-reported autoimmune conditions and the risk of non-Hodgkin lymphoma (NHL) from the InterLymph Consortium demonstrated that a personal history of haemolytic anaemia was associated with an increase in the risk for NHL [odds ratio (OR), 2.57; 95 % confidence interval (CI) 1.27–5.21] [5]. In a multivariate hierarchical regression model, a population-based case–control study in Scandinavia showed a history of AIHA was non-significantly associated with an increased risk of Hodgkin’s lymphoma with an OR of 4.5 (95 % CI, 0.8–24.7) [7].

It has been well-documented that patients with an autoimmune disease (including idiopathic AIHA) have an increased risk of malignancy although the mechanisms are still not completely clear. The underlying autoimmune disorder, with altered lymphocyte reactivity against self- or exogenous antigens are suspected to be the main cause [8–10]. Autoimmune disease may present with secondary AIHA; for example, approximately 5–10 % of patients with systemic lupus erythematosus (SLE) develop the secondary AIHA [9].

Other than idiopathic AIHA, which can be regarded as a disease entity, the remainder of AHA types, including secondary AIHA and the entire group of non-autoimmune AHA (non-AIHA), have various heterogeneous aetiologies. The occurrence of non-AIHA may be a reflection of the characteristics and severity of the parent disease. Patients with non-AIHA caused by any aetiology undergo more or less the same treatments targeting the cause of the haemolysis and its complications, and thus are exposed to potential threats from the use of corticosteroids, blood component transfusion and other therapies which may lead to altered immunity. Little is known about whether patients with non-AIHA have a similar or lesser risk for subsequent development of haematolymphoproliferative malignancies.

We postulated that there is an association between AHA and an increased risk of malignancy in the future. Moreover, there is yet a missing piece of information regarding the estimation of the risk for malignancy, particularly solid tumours, in patients with AHA. Therefore, we conducted a nationwide population-based retrospective cohort study on patients hospitalised for AHA and their subsequent cancer risk. We further differentiated the cancer risk between patients with AIHA and non-AIHA.

## Methods

### Data source

The Taiwan National Health Insurance program has been a single-payer and universal insurance program since 1995 and has enrolled almost 99 % of the citizens of Taiwan in 2007. The Taiwan Ministry of Health and Welfare authorized the National Health Research Institutes (NHRI) to manage all registration files and claims data and to establish the National Health Insurance Research Database (NHIRD). The NHRI created a scrambled and anonymous identification number for each insured person for linking files and to protect the privacy of patients. The NHRI gives the permission to access the data for qualified researchers. The authors have published several population-based studies on the risk of cancer in various clinical settings using the NHIRD [11–15]. This study was conducted after the approval by the Research Ethics Committee of the China Medical University, Taichung, Taiwan (CMU-REC-101-012). This study was performed in accordance with the ethical standards of the 1964 Declaration of Helsinki. The informed consent was waived by the Research Ethics Committee.

For the purpose of research, specific data subsets were constructed for more timely distribution. Three relevant data subsets were chosen for this population-based study, namely, the Registry for Beneficiaries which contained each insured individual’s registration data such as gender, date of birth, occupation and coverage period; the Inpatient Expenditures by Admission which included original claim data of all inpatients and finally, the Registry for Catastrophic Illness Patient Dataset (RCIPD), a unique subset of the NHIRD. Inclusion in the RCIPD required pathologic proof of malignancy, and when in doubt, the application would be examined by an independent haematologist/oncologist medical expense reviewer. Patients who satisfied the criteria for the RCIPD can benefit from a considerable reduction in out-of-pocket expenses for their cancer care throughout the country; this may also create a second check-point control from the patient side to prevent under-reporting of cancer occurrence in the RCIPD. In this research, the disease history was assembled from the Inpatient file. The disease diagnosis was recorded as per the International Classification of Diseases, Ninth Revision, Clinical Modification (ICD–9-CM).

### Study population

**Fig. 1:**
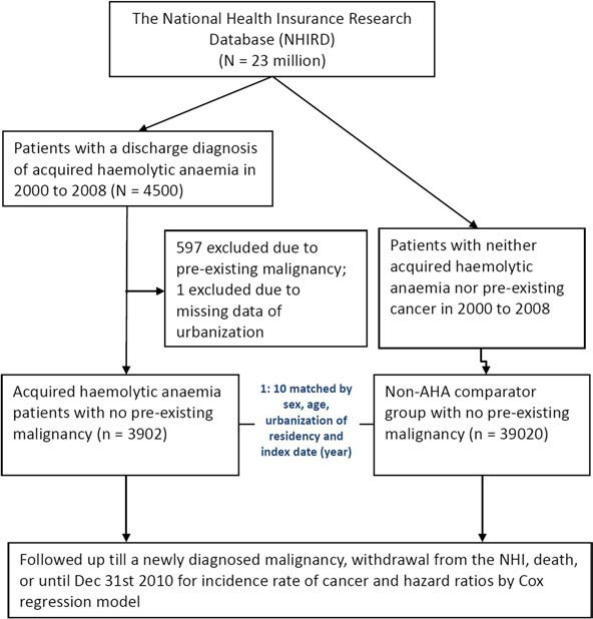
Study flowchart showing steps for the selection of target populations, exclusion criteria and matching of the comparison cohort in the nationwide population-based cohort study

We organized a population-based retrospective cohort study to investigate the association between AHA and subsequent cancer risk. The flow chart of the study population selection is shown in Fig. 1. The AHA cohort consisted of patients with newly-diagnosed AHA (ICD-9-CM 283, from the inpatient records from 2000 to 2008. The index date was set at six months after (the first episode if more than one) the diagnosis of AHA was given at the hospital discharge. Patients with pre-existing malignancy before the index date were excluded. The AHA patients were separated into two sub-cohorts: a non-AIHA sub-cohort (ICD-9-CM codes, 283.1, 283.2 and 283.9) and an AIHA sub-cohort (ICD-9-CM code = 283.0). Because of the nature of an ICD-9-CM diagnosis categorization, a drug-induced haemolytic event in a patient with glucose-6-phosphate dehydrogenase (G6PD) deficiency would be coded as inherited haemolytic anaemia and thus was not included in the studied cohort of AHA.

The comparison cohort comprised individuals who had not been diagnosed with AHA or pre-existing cancer from 2000 to 2008. In order to increase the statistical power, for each AHA patient, we randomly selected 10 comparison persons from the general population frequency-matched by age (per 5 years), gender, urbanization of residency and index date. The index date of the comparison person was randomly matched by the same index year as that of the AHA case. The comparison cohort also had the same half year of lag observation time. We excluded all individuals with cancer diagnosed before the index date. The main parameter under consideration in this study was the incidence of developing cancer (ICD-9-CM 140–208, from the RCPID). The follow-up was terminated when cancer developed, or censored when the patient withdrew from the insurance, lost to follow-up or deceased, or on 31st December 2010 (Fig. 1).

The study also collected the co-morbidity history for each study subject as confounding factors. The co-morbidities before the index date included diabetes mellitus (DM, ICD-9-CM 250), alcohol use disorders (ALD, ICD-9-CM 265.2, 291, 303, 305.0, 357.5, 425.5, 535.3, 571.0, 571.1, 571.2, 571.3, 980.0 and V11.3), chronic kidney disease (CKD, ICD-9-CM 585), splenomegaly (ICD-9-CM 289.4), liver cirrhosis (ICD-9-CM 571.2, 571.5 and 571.6), hepatitis B virus infection (HBV, ICD-9-CM 070.2, 070.3 and V02.61) and hepatitis C virus infection (HCV, ICD-9-CM V02.62, 070.41, 070.44, 040.51 and 070.54) from the inpatient file and systemic lupus erythematosus (SLE, ICD-9-CM 710.0) and rheumatoid arthritis (RA, ICD-9-CM 714) from the RCPID. The urbanization level of residency was based on several index including population density (people/km^2^), and population ratio of different educational levels, population ratio of elderly, population ratio of people of agriculture workers and the number of physicians per 100,000 people [16]. We categorized the urbanization of residency into 4 levels. The level 1 indicated the highest urbanization level and the level 4+ meant the lowest level.

### Statistical analysis

We compared distributions of age group, gender and co-morbidities and the mean and standard deviation (SD) for age between AHA and comparison cohorts. We calculated the overall incidence density rates of cancer for both cohorts, using the total number of cancer events divided by the total sum of follow-up years for each cohort. Cox proportional hazards regression analysis was used to estimate the AHA cohort to the comparison cohort hazard ratio (HR) and 95 % confidence interval. Multivariable Cox model was used to calculate the adjusted hazard ratio (aHR) and 95 % confidence interval including sex, age, urbanization of residency and all comorbidities in the model. Further data analysis calculated the incidence of individual cancer and the related AHA cohort to comparison cohort aHR for major cancers, including cancers of lung (ICD-9-CM 162), liver (ICD-9-CM 155), colorectal (ICD-9-CM 153 and 154), breast (ICD-9-CM 174, only in female), lymphatic and hematopoietic tissue(ICD-9-CM 200–208) and others. We also used Kaplan-Meier method to measure and plot the cumulative incidence for both cohorts and used log-rank test to examine the difference between the 2 cohorts. The proportional hazards assumption was not violated in the scaled Schoenfeld residuals test (*p =* 0.21). SAS 9.3 software (SAS Institute, Cary, NC, USA) was used to manage and analyse the data. The cumulative incidence curve was plotted by SPSS. The significant level was set at less than 0.05 for two-side testing of *p-*value.

## Results

We finally enrolled 3,902 AHA patients and 39,020 healthy individuals for comparison with similar mean age (42 years) and sex ratio (male: 43 %) (*p* > 0.05) in this study (Table 1). The proportion of AIHA in the AHA cohort was 32 %.

**Table 1:** Baseline demographic data and comorbidity compared between the comparison and the acquired haemolytic anaemia (AHA) cohorts

Variable	Comparison cohort *n =* 39020 (%)	AHA cohort *n =* 3902 (%)
Age, years (SD)	42.3 (25.4)	42.3 (25.5)
< 20	8920 (22.9)	892 (22.9)
20–39	9200 (23.6)	920 (23.6)
≥ 40	20900 (53.6)	2090 (53.6)
Sex		
Female	22230 (57.0)	2223 (57.0)
Male	16790 (43.0)	1679 (43.0)
Urbanization of residency		
1 (highest)	9360 (24.0)	936 (24.0)
2	12990 (33.3)	1299 (33.3)
3	6260 (16.0)	626 (16.0)
4+ (lowest)	10410 (26.7)	1041 (26.7)
Type of AHA		
Autoimmune	0	1246 (31.9)
Non-immune	0	2656 (68.1)
Comorbidity		
Diabetes	2716 (7.0)	499 (12.8)
SLE	18 (0.05)	263 (6.7)
Alcohol-use disorder	147 (0.4)	76 (1.9)
Splenomegaly	25 (0.1)	130 (3.3)
CKD	171 (0.4)	247 (6.3)
Liver cirrhosis	161 (0.4)	139 (3.6)
RA	63 (0.2)	37 (0.9)
HBV	108 (0.3)	72 (1.8)
HCV	99 (0.3)	94 (2.4)

*Abbreviations: AHA* acquired haemolytic anaemia, *CKD* chronic kidney disease, *HBV* hepatitis B virus infection, *HCV* hepatitis C virus infection, *RA* rheumatoid arthritis, *SD* standard deviation, *SLE* systemic lupus erythematosus

Amongst the patients with AHA, 13 % had diabetes mellitus, 7 % had SLE and 6 % had chronic kidney disease. The proportions of the comorbidities in AHA cohort were higher than the proportions in comparison cohort (*p* < 0.0001).

**Fig. 2:**
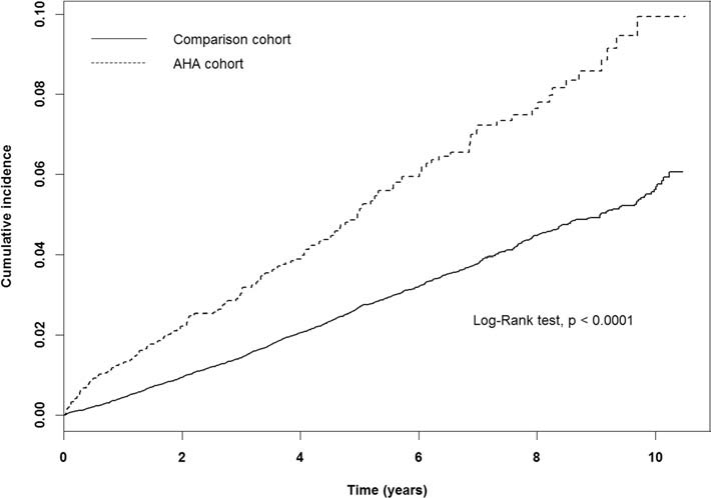
The cumulative incidence of cancer in the study cohorts

The cumulative incidence of cancer after 11-year follow-up measured by Kaplan-Meier method was 3.9 % greater in the AHA cohort than in the comparison cohort (log-rank test, *p* < 0.0001; Fig. 2).

During a total of 17,912 patient-years for the AHA cohort under observation, 187 cancers occurred. Table 2 shows that the incidence density of cancer was 1.9-fold greater in the AHA cohort than in the comparison cohort (104 vs. 54.7 per 10,000 person-years) with an adjusted HR of 1.78 (95 % CI = 1.56–2.59) in the multivariable Cox proportional hazards regression analysis. Regarding the subtypes, compared with the individual without AHA, there were increased hazard of developing cancer for both non-AIHA patients (HR = 1.87, 95 % CI = 1.53–2.29) and AIHA patients (HR = 2.01, 95 % CI = 1.56–2.59), respectively. In this study, male gender [adjusted HR (aHR) 1.50, 95 % CI = 1.34–1.67)], DM (1.29, 1.10–1.51), CKD (1.54, 1.08–2.21), liver cirrhosis (1.96, 1.30–2.94) and infection with HCV (2.78, 1.89–4.08) were significantly associated with an increased risk of cancer.

**Table 2:** Incidence of cancer and stratified analysis with adjusted hazard ratios by multivariate Cox proportional hazards regression analysis for study cohort

Variable	Event	PYs	Rate	Crude HR (95 % CI)	Adjusted HR (95 % CI)
AHA					
No	1130	206599	54.7	ref	ref
Yes	187	17912	104	1.92(1.64–2.24)	1.78(1.50–2.12)
Non-Autoimmune AHA	119	12107	98.3	1.81(1.50–2.19)	1.87(1.53–2.29)
Autoimmune AHA	68	5805	117	2.15(1.68–2.75)	2.01(1.56–2.59)
Age group					
< 25	9	55421	1.62	ref	ref
25–39	54	52892	10.2	6.32(3.12–12.8)	6.78(3.35–13.7)
≥ 40	1254	116197	108	66.9(34.7–129)	65.37(33.9–126)
Sex					
Female	606	127460	47.5	ref	ref
Male	711	97051	73.3	1.54(1.38–1.71)	1.50(1.34–1.67)
Urbanization of residency					
1 (highest)	282	53113	53.1	ref	ref
2	446	77556	57.5	1.08(0.93–1.25)	0.99(0.85–1.14)
3	185	34097	54.3	1.02(0.85–1.23)	1.12(0.93–1.34)
4+ (lowest)	404	59745	67.6	1.27(1.09–1.48)	1.08(0.93–1.26)
Comorbidity					
DM					
No	1128	210752	53.5	ref	ref
Yes	189	13759	137	2.61(2.24–3.05)	1.29(1.10–1.51)
SLE					
No	1309	223337	58.6	ref	ref
Yes	8	1174	68.2	1.18(0.59–2.37)	1.04(0.51–2.12)
Alcohol^a^					
No	1297	223606	58.0	ref	ref
Yes	20	905	221	3.88(2.49–6.04)	1.09(0.66–1.81)
Splenomegaly					
No	1303	223914	58.2	ref	ref
Yes	14	597	235	4.10(2.42–6.94)	1.50(0.85–2.66)
CKD					
No	1284	223018	57.6	ref	ref
Yes	33	1493	221	3.91(2.77–5.52)	1.54(1.08–2.21)
Liver cirrhosis					
No	1278	223469	57.2	ref	ref
Yes	39	1042	374	6.71(4.88–9.24)	1.96(1.30–2.94)
RA					
No	1314	224077	58.6	ref	ref
Yes	3	434	69.1	1.2(0.39–3.71)	0.79(0.25–2.47)
HBV					
No	1303	223738	58.2	ref	ref
Yes	14	773	181	3.15(1.86–5.33)	1.38(0.80–2.38)
HCV					
No	1286	223814	57.5	ref	ref
Yes	31	697	445	7.99(5.59–11.4)	2.78(1.89–4.08)

Adjusted model was mutually adjusted

*Abbreviations: AHA* acquired haemolytic anaemia, *Alcoho*l^a^ alcohol-use disorders, *CI* confidence interval, *CKD* chronic kidney disease, *HBV* hepatitis B virus infection, *HCV* hepatitis C virus infection, *HR* hazard ratio, *PYs* person-years, *Rate* incidence rate, per 10,000 person-years, *RA* rheumatoid arthritis, *ref* reference, *SLE* systemic lupus erythematosus

Table 3 shows the development of different types of cancer between the AHA and comparison cohorts. Overall, relative to the individuals without AHA, the patients with AHA were significantly associated with an increased risk of lymphatic and haematopoietic (HR = 13.1, 95 % CI = 8.46–20.3) and other malignant solid tumours (HR = 1.82, 95 % CI = 1.40–2.35). Patients with non-AIHA and AIHA had near 10-fold (HR = 9.59, 95 % CI = 5.57–16.5) and 20-fold (HR = 19.5, 95 % CI = 11.5–32.8) increased risk of lymphatic and haematopoietic tumours, respectively.

**Table 3:** Incidence of different types of cancer and measured hazard ratios by multivariate Cox proportional hazards regression analysis for study cohorts

Cancer type	Liver	Lung	Colorectal	Lymphatic and haematopoietic tissue	Breast	Others
	HR (95 % CI)	HR (95 % CI)	HR (95 % CI)	HR (95 % CI)	HR (95 % CI)	HR (95 % CI)
Comparison cohort	ref	ref	ref	ref	ref	ref
AHA cohort	1.23(0.77–1.98)	1.28(0.73–2.23)	1.16(0.65–2.07)	13.1(8.46–20.3)	1.13(0.55–2.30)	1.82(1.40–2.35)
Non-AIHA	1.43(0.84–2.43)	1.24(0.62–2.46)	1.28(0.65–2.50)	9.59(5.57–16.5)	1.23(0.53–2.88)	1.81(1.34–2.46)
AIHA	0.91(0.41–2.01)	1.35(0.55–3.30)	0.95(0.35–2.61)	19.5(11.5–32.8)	0.98(0.34–2.85)	1.82(1.22–2.73)

Model adjusted for age, sex, urbanization of residency, DM, SLE, alcohol-use disorders, splenomegaly, CKD, liver cirrhosis, HBV, HCV and RA

*Abbreviations: AHA* acquired haemolytic anaemia, *CKD* chronic kidney disease, *DM* diabetes mellitus, *HBV* hepatitis B virus infection, *HCV* hepatitis C virus infection, *RA* rheumatoid arthritis, *ref* reference, *SLE* Systemic Lupus Erythematosus

Table 4 shows the sensitivity analysis conducted for the risk of cancer between the AHA and comparison cohorts by follow-up years. The results suggested that patients with AHA were associated with a significantly increased risk of developing cancer as compared with individuals without AHA, although all of the study population had at least four years of follow-up.

**Table 4:** Sensitivity analysis showing varying estimates of the adjusted risk of developing subsequent cancer utilizing a Cox model by different cut-offs in lengthening the time lag for follow-up

	Comparison cohort			AHA cohort			Crude HR (95 % CI)	Adjusted HR (95 % CI)
Variable	Event	PYs	rate	Event	PYs	rate		
Time lag (year)								
> 1	961	206171	46.6	138	17765	77.7	1.70(1.42–2.04)	1.69(1.39–2.05)
> 2	771	200747	38.4	106	17131	61.9	1.67(1.36–2.04)	1.68(1.35–2.10)
> 3	610	187711	32.5	82	15680	52.3	1.66(1.32–2.09)	1.70(1.32–2.19)
> 4	433	170395	25.4	61	13926	43.8	1.78(1.36–2.33)	1.75(1.30–2.36)

Model adjusted for age, sex, urbanization of residency, DM, SLE, alcohol-use disorders, splenomegaly, CKD, liver cirrhosis, HBV, HCV and RA

*Abbreviations: AHA* acquired haemolytic anaemia, *CI* confidence interval, *HR* hazard ratio, *PYs* person-years, *rate* incidence rate, per 10,000 person-years

## Discussion

This nationwide population-based retrospective frequency-matched cohort study with 17,919 patient-years follow-up for the entire AHA cohort had an approximately 80 % increase in the hazard for subsequent malignancy as compared with the non-AHA comparators. The randomly-selected comparison cohort was matched for age, gender, urbanization of residency and index date. Confounding factors such as type 2 diabetes, alcohol-use disorder, splenomegaly, chronic kidney disease, rheumatoid arthritis, history of viral hepatitis B or C infection and liver cirrhosis were adjusted in the construction of the Cox model. Not only did the risk increase in patients with AIHA (aHR = 2.01) but it also increased in patients with non-AIHA (aHR = 1.87) that had resulted from a group of heterogeneous aetiologies. To the best of our knowledge, this study provided for the first time the evidence for and the best estimate of the risk for subsequent cancer in patients with non-autoimmune haemolytic anaemia.

In this study, the sensitivity analysis conducted to help understand whether the risk would still persist after up to four years of follow-up demonstrated that the aHR was still around 1.75 beyond the fifth year of the follow-up. This sensitivity analysis helped exclude the possibility of protopathic bias (reverse causation) because some haematolymphoproliferative disorders may go unnoticed for years. In the literature, a pooled analysis of the InterLymph Consortium accrued 29,423 participants from 12 case–control studies and computed the pooled odds ratios at 2.5 (95 % CI, 1.08–5.83) in a joint fixed-effect model for the future development of non-Hodgkin’s lymphoma ten years after a self-reported history of haemolytic anaemia [5]. It has been noted that the use of self-reported history of haemolytic anaemia had an inherent risk for exposure misclassification bias.

The risk for lymphatic-haematopoietic malignancy was increased in both the AIHA (19.5-fold) and non-AIHA (9.6-fold) sub-cohorts in this study. Anderson et al. reported the magnitude of the association in terms of odds ratio of AIHA and chronic myeloproliferative disorder (CMPD) (excluding chronic myeloid leukaemia) to be 11.9 (4.72–30.2); however, after excluding claims within 5 years of CMPD diagnosis, the OR became statistically insignificant (OR, 4.02; 95 % CI, 0.50–32.5) [17]. In the same study, they also demonstrated a significant association between AIHA and acute myeloid leukaemia (OR, 3.74; 95 % CI, 1.94–7.22), chronic myeloid leukaemia (OR, 5.23; 95 % CI, 1.82–15.0), and myelodysplastic syndromes (OR, 4.12; 95 % CI, 1.66–10.2).

This study also revealed that the risk for lymphatic-haematopoietic malignancies and for certain malignant solid tumours increased. Individuals of the entire AHA cohort had an increased risk for solid tumours other than those occurring in the liver, lung, colorectal and breast.

The linkage datasets methodology utilized in the investigation of cancer incidence in this cohort study produced robust and reliable results because of the use of the unique registry dataset of severe illnesses such as cancer that offered a second check mechanism for cancer diagnosis ascertainment. It is noteworthy that this study also captured the outcomes of the risk for subsequent total cancer occurrence in individuals with diabetes mellitus (aHR 1.29; range, 1.10–1.51), chronic kidney disease (aHR 1.54; range, 1.08–2.21), liver cirrhosis (aHR 1.96; range, 1.30–2.94), and HCV infection (aHR 2.78; range, 1.89–4.08), which were in-line with the current understanding of these risks from the literature [18–22].

It may be an oversimplification to attribute the mechanisms for the positive association with future malignancies in patients with non-AIHA solely to the most feared complication resulting from prior exposure to corticosteroid therapy for controlling haemolytic anaemia and its underlying systemic disorder. The authors speculated that perhaps haemolysis itself will alter the circulating concentrations of angiogenic and pro-inflammatory markers which could contribute to the increased cancer risk [23–25].

Utilizing the coding from the discharge diagnoses to capture the occurrence of AHA and medical co-morbidities has been regarded as more reliable than the use of the outpatient billing records because billings using the discharge diagnoses will go through the hands of qualified medical coding specialists [26]. Nevertheless, the potentials for inaccurate ICD-9-CM coding may exist for any administrative claims-based research. A few of the limitations of this study must be noted. Owing to the de-identified nature of each claim record in the datasets, a chart review of the patient’s medical record was not possible. In addition, the datasets from the NHIRD did not contain biological data such as height, weight and smoking history or serial hemogram data so that the severity of haemolytic anaemia could not be determined. These limitations may potentially affect the risk estimates in this study.

## Conclusions

In conclusion, the adjusted hazard ratio for lymphatic-haematopoietic malignancy was elevated for 20-fold in the AIHA group and for 10-fold in the non-AIHA group. This study also provided the risk estimates for future solid tumour occurrence in patients with acquired haemolytic anaemia, particularly of malignant solid tumours other than those occurring in the lung, colorectum, liver and breast (80 % increased risk).

## Abbreviations

AHA: acquired haemolytic anaemia
aHR: adjusted hazard ratio
AIHA: autoimmune haemolytic anaemia
CKD: chronic kidney disease
HBV: hepatitis B virus
HCV: hepatitis C virus
ICD-9-CM: international classification of diseases, ninth edition, clinical modifications
NHIRD: National Health Insurance Research Database
NHRI: National Health Research Institute Taiwan
Non-AIHA: non-autoimmune haemolytic anaemia
OR: odds ratio
PNH: paroxysmal nocturnal haemoglobinuria
RA: rheumatoid arthritis
RCPID: Registry for Catastrophic Illness Patient Dataset
SLE: systemic lupus erythematosus
